# Supplementary material for: Proximal and fatty acid analysis in Ostrea chilensis, Crassostrea gigas and Mytilus chilensis (Bivalvia: Mollusca) from southern Chile
Source: PLoS One. 2022 Jul 7;17(7):e0270825. doi: 10.1371/journal.pone.0270825 (PMC9262220; doi:10.1371/journal.pone.0270825)
Supplement: Attachment — Submitted filename: Comment_toRevision1_Attachment.pdf [file pone.0270825.s002.pdf]

Here are my comments on some of the authors' responses. I have stopped further review of the text. I will be happy to look at it again after an appropriate revision.

All the best

Line 26/27: Please clarify the definitions of equal contribution of the authors - ¶ vs. &.

Reply: the authors' equal contribution to:

¶ = all of these authors contributed to study design, data collection and analysis, decision to publish, or manuscript preparation.

&= all these authors contributed to the experimental and field work.

- Thank you, but this information should also be in the paper, shouldn't it?

Line 83: Change into "On the one hand…"

Reply: done.

- hm,, the authors revised the sentence but not as suggested. That's fine but the reply "done" is irritating.

Line 107f: Sampling of animals: Please clarify the sentence "3 samples of 20 oysters" as it is 60 animals in total, isn't it? Please add more details about the environmental parameters at the time of collection (water temperature, salinity...) and transport of the animals (duration, transport on ice/water...). Were the animals kept in institutional aquarium systems prior to dissection? If yes, please add details on husbandry.

Reply: For each locality, 60 sampled specimens were collected from cultivation areas, where 3 samples (replicates) of 20 individuals were randomly made, except for C. gigas, which were fewer specimens (40 individuals). Then, in the laboratory, the tissues without the shell were worked, each sample was stored in a refrigerated/frozen ziplox bag with the meat of 20 individuals corresponding to replica 1, 2 and 3 of each sampling.

- "Please add more details…." This comment wasn't addressed, neither in the reply nor in the revised ms.

Line 121ff: Please comment on using the calculation of yield instead of condition index (soft body dry mass*100/shell dry mass) which is more common. In any case, data sets should be compared with literature data to increase the reliability of the results.

Reply: The samples were processed in the field, where the wet weight was directly measured. In our laboratory, the samples were lyophilized to be considered for proxima content and fatty acid content. The work of Sing & Ransangan (2019) measure yield according to wet weight.

Sing, O. F., & Ransangan, J. (2019). Effect of physicochemical parameters and phytoplankton composition on growth performance of green mussel (Perna viridis) in

- Why don't the authors provide this citation in the ms? Will the graphs be found in the supplements?

Line 133: Please clarify how samples were treated prior to analysis to ensure homogeneity, as parameters were determined in whole animal extracts.

Reply: For each sample of 20 individuals the tissues were pooled and freeze dried in a Savant freeze dryer (-80°C), then they were ground to homogenize and saved at -41°C until the respective biochemical analysis.

- This is an important information that the tissue of 20 individuals was pooled prior to analyses and must be given in the ms.

Line 133: Unclear, please add more information how crude protein was calculated.

Reply: In nutrition, the total nitrogen content of the sample is converted into a value called "crude protein" by multiplying it by 6.25, a constant based on the conventional assumption that any protein is composed of 16% nitrogen.

- Again, this is an information that should be given in the ms.

Line 146f: Repetition, see line 131f.

Reply: corrected

- Sorry, the repetition is still in the revised version.
