# Supplementary material for: Proximal and fatty acid analysis in Ostrea chilensis, Crassostrea gigas and Mytilus chilensis (Bivalvia: Mollusca) from southern Chile
Source: PLoS One. 2022 Jul 7;17(7):e0270825. doi: 10.1371/journal.pone.0270825 (PMC9262220; doi:10.1371/journal.pone.0270825)
Supplement: Attachment — Submitted filename: Response to Reviewers.doc [file pone.0270825.s003.doc]

REPLIES TO REVIEWER

PONE-D-21-28284

Proximal and fatty acid analysis in *Ostrea chilensis*, *Crassostrea gigas* and *Mytilus chilensis* (Bivalvia: Mollusca) from Southern Chile.

Dear Academic Editor

Below you can find the corrections made in the manuscript according to the suggestions made by the reviewer # 2:

**REVIEWER #2 COMMENTS:**

Line 26/27: Please clarify the definitions of equal contribution of the authors - ¶ vs. &.

Reply: the authors' equal contribution to:

¶ = all of these authors contributed to study design, data collection and analysis, decision to publish, or manuscript preparation.

&= all these authors contributed to the experimental and field work.

- Thank you, but this information should also be in the paper, shouldn't it?

Reply: We appreciate your clarification. Your suggestion will be included in the manuscript.

Line 83: Change into “On the one hand…”

Reply: done.

- hm,, the authors revised the sentence but not as suggested. That's fine but the reply "done" is irritating.

Reply: We regret the incorrect use of the word "done". We will take this into account in future corrections.

Line 107f: Sampling of animals: Please clarify the sentence "3 samples of 20 oysters" as it is 60 animals in total, isn't it? Please add more details about the environmental parameters at the time of collection (water temperature, salinity...) and transport of the animals (duration, transport on ice/water...). Were the animals kept in institutional aquarium systems prior to dissection? If yes, please add details on husbandry.

Reply: For each locality, 60 sampled specimens were collected from cultivation areas, where 3 samples (replicates) of 20 individuals were randomly made, except for C. gigas, which were fewer specimens (40 individuals). Then, in the laboratory, the tissues without the shell were worked, each sample was stored in a refrigerated/frozen ziplox bag with the meat of 20 individuals corresponding to replica 1, 2 and 3 of each sampling.

- Please add more details….” This comment wasn't addressed, neither in the reply nor in the revised ms.

Reply: We improved the paragraph, including the required information in the manuscript. We appreciate your helpful suggestions.

Line 121ff: Please comment on using the calculation of yield instead of condition index (soft body dry mass*100/shell dry mass) which is more common. In any case, data sets should be compared with literature data to increase the reliability of the results.

Reply: Samples were processed in the field, where 20 individuals were randomly selected for each sample, measured and weighed wet, shells were immediately removed and the soft bodies of the 20 individuals were grouped in a properly labeled bag. The bags were stored cold for transport to the laboratory. In the laboratory, the samples were frozen, freeze-dried, macerated and grouped by bag, kept frozen until analysis for proximal and fatty acid content.

The index was calculated according to Hickmann and Illingworth (1980).

These authors compared different condition index formulas that are weight/weight ratios and that reflect changes in body proportions in bivalve populations.

CI_wet_ = [wet meat volumen/(whole volumen - Shell volumen)] * 100

CI_volume_ = [dry meat wt / (whole volumen - shell volumen)] * 100

CI_weight_ = [dry meat wt/(whole wt - shell wt)] * 100

CI_commercial_ = [Meat wet wt / Total wet weight] * 100

_They found that_ CI_commercial correlated 92.7% with CIwet, 90.4% with_ CI_volume and 90.9% with CIweight._

According to Hickmann and Illingworth (1980), although CIweight is the most advisable for reliable comparisons, it is possible to standardize the measurement of wet weight in an index such as CIcommercial to minimize errors by always adopting the same weighing routines, which is what was applied in this study.

Line 133: Please clarify how samples were treated prior to analysis to ensure homogeneity, as parameters were determined in whole animal extracts.

Reply: For each sample of 20 individuals the tissues were pooled and freeze dried in a Savant freeze dryer (-80°C), then they were ground to homogenize and stored at -41°C until the respective biochemical analysis.

- This is an important information that the tissue of 20 individuals was pooled prior to analyses and must be given in the ms.

Reply: Thank you for the clarification. This information has been included in the manuscript.

Line 133: Unclear, please add more information how crude protein was calculated.

Reply: In nutrition, the total nitrogen content of the sample was converted into a value called "crude protein" by multiplying it by 6.25, a constant based on the conventional assumption that any protein is composed of 16% nitrogen.

- Again, this is an information that should be given in the ms.

Reply: Thank you for the clarification. This information has also been included in the manuscript.

Line 146f: Repetition, see line 131f.

Reply: corrected

- Sorry, the repetition is still in the revised version.

Reply: This repetition has been removed. Thanks for the clarification
